# Visual Tracking and Organ Targeting of *Naja atra* and *Deinagkistrodon acutus* Venoms in Mice

**DOI:** 10.3390/toxins17110559

**Published:** 2025-11-13

**Authors:** Shaocong Hu, Manqi Xiao, Ningjing Jiang, Ziyan Zhang, Qiuju Jia, Yi Zhou, Xin Liu, Ming Liao

**Affiliations:** 1 Life Science Institute, Guangxi Medical University, Nanning 530021, China; shaocong_hu@163.com (S.H.);; 2 Clinical Medical Laboratory, Air Force Medical Center, PLA, Beijing 100142, China

**Keywords:** *Naja atra*, *Deinagkistrodon acutus*, CY7-SE, visual tracking, organ targeting

## Abstract

In China, bites caused by the *Naja atra* and *Deinagkistrodona acutus* are the most common types of snakebites. While the functional characteristics of the two venom components have been well documented, their in vivo metabolic pathways, target organ distribution patterns, and dynamic pharmacokinetic profiles remain less explored. This study established a murine envenoming model through CY7-SE labeling of *Naja atra* and *Deinagkistrodon acutus* venoms. The real-time in vivo absorption and biodistribution of venoms were dynamically monitored via fluorescence imaging, with subsequent proteomic profiling to characterize organ-specific toxin targeting patterns. Gel filtration chromatography and HPLC analyses validated labeling efficiency at ratios of 0.1 mg CY7-SE per 1 mg *Naja atra* venom and 0.075 mg CY7-SE per 1 mg *Deinagkistrodon acutus* venom, with electrophoretic confirmation of protein integrity and preserved 740 nm fluorescence excitation. Acute toxicity assays demonstrated no significant difference in LD_50_ lethality between labeled and native venoms (*p* > 0.05). The intoxication models revealed species-specific pathophenotypes, i.e., CY7-*Naja atra* venom induced systemic weakness, tachypnea, and inflammatory necrosis in lung, myocardium, and liver, whereas CY7-*Deinagkistrodon acutus* venom provoked hemorrhagic diathesis. Both models exhibited marked leukocytosis, transaminitis, and elevated creatinine levels (*p* < 0.05). Fluorescence tracing uncovered distinct biodistribution kinetics: *Deinagkistrodon acutus* venom achieved peak organ accumulation at 3 h with rapid dissemination (24 h injection-site retention: 12.61%), contrasting with *Naja atra* venom’s delayed 6 h peak and prolonged renal sequestration (24 h injection-site retention: 60.9%). Target organ proteomic profiling identified *Deinagkistrodon acutus*-enriched thrombin-like enzymes and metalloproteinases in lung/liver/spleen, while *Naja atra* venom predominantly accumulated renal acidic phospholipase A_2_ and weakly neurotoxic NNAM2.

## 1. Introduction

Snakebite envenoming, classified by the World Health Organization (WHO) as a neglected tropical disease, poses a significant global public health burden. WHO estimates suggest approximately 5 million snakebite incidents occur annually worldwide, leading to 100,000 fatalities and 400,000 cases of permanent disability [1,2].

There are about 60 kinds of venomous snakes in China, with the clinical bites of *Naja atra* and *Deinagkistrodon acutus* ranking at the top [3]. Snake venom is usually a mixture composed of 20–100 substances, of which more than 90% are proteins [4], and the remaining parts mainly include carbohydrates, lipids, amines, and inorganic salts [5,6,7]. The venom of *Naja atra* is mainly composed of phospholipase A2 (PLA_2_, 12.2%), metalloproteinase (SVMP, 1.6%), cysteine-rich secretory protein family (CRISP, 1.8%), and three-finger toxin (3FTx, 84.3%) [8]. The venom of sharp-nosed viper mainly consists of PLA_2_ (7.3%), snake venom serine protease (SVSP, 6.6%), SVMP (46.9%), C-type lectin-like protein (SNACLEC, 37.6%), L-amino acid oxidase (LAAO) and CRISP (1.6%) [9].

After a biological organism is bitten by a snake, the venom is absorbed into various different circulatory systems of the body and targets specific tissues, organs, or systems, thereby causing pathological changes in the body, different types of snakes and venom components lead to a series of different clinical changes [10,11]. For example, circulatory system disorders caused by viper bites, and respiratory system disorders caused by cobra bites [12,13]. The bite of *Naja atra* is a mixed type of poisoning. A serious complication after snake venom is injected into the body is the necrosis of local muscle tissue near the wound. The wound often forms ulcers that are difficult to heal, leading to limb disabilities in many patients, and in severe cases, amputation may even be required [14,15]. The bite of *Deinagkistrodon acutus* is a hemotoxic type of envenoming. The clinical symptoms of the bite generally manifest as persistent severe pain at the wound site and continuous bleeding. The tissue at the wound site is prone to necrosis and ulceration, with subcutaneous bleeding, purpura, proteinuria, and abnormal liver and kidney function [16]. The existing clinical detection techniques currently cannot timely and effectively determine the nature and content of snake venom toxins in patients’ bodies, including those from *Naja atra* and *Deinagkistrodon acutus* bites, which brings great difficulties to clinical diagnosis and treatment. However, the dynamic changes in the absorption, distribution, targeting, and elimination of snake venom in the body and the patterns of time changes are crucial for understanding the pathophysiology of snakebite, formulating snakebite risk assessment strategies, and improving the treatment plans for snakebite [7]. Currently, most methods employ ELISA or radioisotope labeling techniques to analyze the absorption, distribution, elimination, and clearance processes of snake venom within biological organisms. The ELISA method requires the preparation of monoclonal antibodies, while the radioisotope method faces challenges such as uncontrollable radioactive elements and short half-lives [17,18,19]. Therefore, the field of snake venom research urgently requires a new method capable of tracking the distribution of exogenous venom within the body, its target sites, and the dynamic changes in the organism’s clearance of foreign toxins. Fluorescent labeling has become a cornerstone of biomedical research owing to its exceptional sensitivity, specificity, and quantitation capabilities [20,21]. Cyanine dyes represent high-performance molecular tracers, characterized by ultrahigh molar extinction coefficients (>150,000 M^−1^cm^−1^) and quantum efficiencies (Φ > 0.3). Their amine-reactive moieties facilitate stable covalent conjugation with proteins, antibodies, and peptides via straightforward coupling chemistry [17,22]. The Cy7-SE dye employed in this study, featuring two indolenine rings interconnected by a heptamethine chain, utilizes its N-hydroxysuccinimide ester (NHS ester) group to form durable amide bonds (bond energy > 300 kJ/mol) with primary amines on target proteins, thereby providing a robust platform for venom protein labeling.

The near-infrared spectral range (650–900 nm) in mammalian tissues offers minimal autofluorescence (<5% background intensity), attenuated light scattering (scattering coefficient < 0.1 mm^−1^), and improved penetration depth (>5 mm) [23]. These optical advantages enable noninvasive in vivo imaging in small animals, allowing real-time monitoring of toxin metabolism and biodistribution with preserved physiological fidelity.

Building on this foundation, we aimed to delineate the in vivo absorption and distribution kinetics of *Naja atra* (Elapidae) and *Deinagkistrodon acutus* (Viperidae) venoms using Cy7-SE-based fluorescent labeling coupled with live-animal imaging. Complementary shotgun proteomics characterized toxin repertoires within high-accumulation organs, clarifying temporal dissemination patterns and organ-specific toxicodynamics. By mapping toxin distribution profiles, this study seeks to inform optimal therapeutic windows for antivenom intervention and advance mechanistic understanding of systemic envenomation. The integrated imaging-proteomics platform developed herein further establishes a transformative framework for investigating venom toxicokinetics, offering novel insights into biodistribution pathways and toxicity cascades.

## 2. Results

### 2.1. Systematic Validation of Fluorescent Labeling Efficiency and Structural Integrity in Snake Venom Proteins via Chromatographic, Electrophoretic, and Optical Modalities

#### 2.1.1. Quantitative Validation of Fluorescent Labeling Efficiency

**Figure 1:**
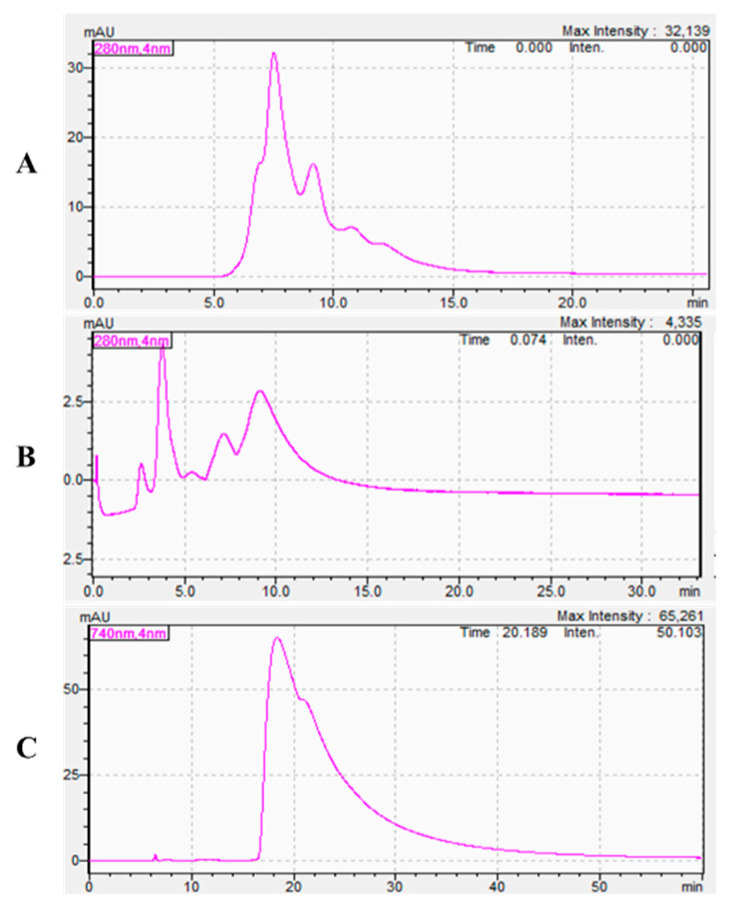
*Naja atra* venom, *Deinagkistrodon acutus* venom, and CY7-SE in Gel Permeation Chromatography Column ((**A**): *Deinagkistrodon acutus* venom; (**B**): *Naja atra* venom; (**C**): CY7-SE).

The molecular sieving effect of gel filtration chromatography columns results in notably higher migration rates for low-mW proteins compared to their high-mW counterparts. As shown in Figure 1, distinct elution profiles were observed between unlabeled *Naja atra* venom (retention time < 15 min) and *Deinagkistrodon acutus* venom (retention time < 15 min), while the characteristic peak of CY7-SE fluorophore emerged after 16 min. This differential separation confirms the effective discrimination between labeled venom proteins and free CY7-SE within the chromatographic system.

#### 2.1.2. Validation of Concentration-Dependent Fluorescent Labeling Mechanisms

**Figure 2:**
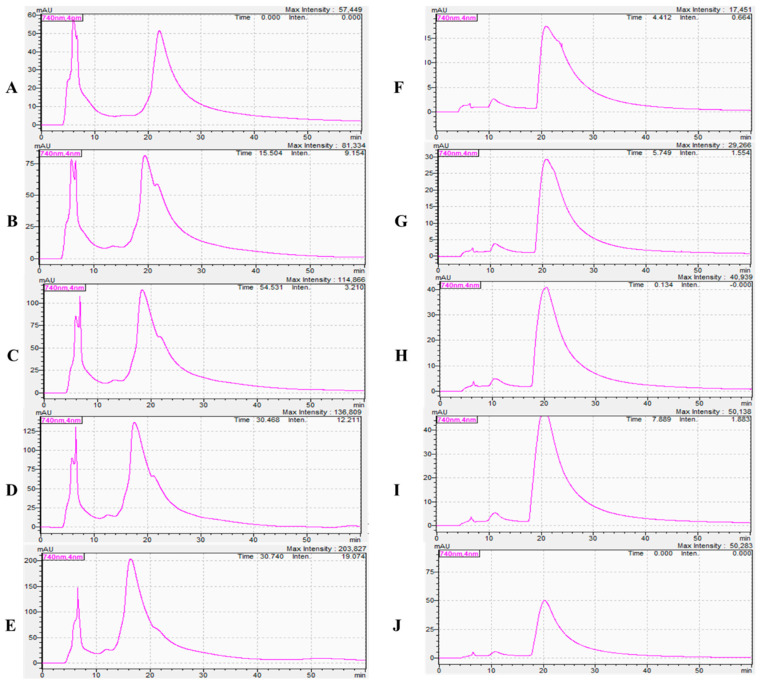
Results of the Gradient Labeling Experiment. ((**A**): 0.05 mg CY7-SE + 1 mg *Deinagkistrodon acutus* venom; (**B**): 0.075 mg CY7-SE + 1 mg *Deinagkistrodon acutus* venom; (**C**): 0.1 mg CY7-SE + 1 mg *Deinagkistrodon acutus* venom; (**D**): 0.125 mg CY7-SE + 1 mg *Deinagkistrodon acutus* venom; (**E**): 0.15 mg CY7-SE + 1 mg *Deinagkistrodon acutus* venom; (**F**): 0.05 mg CY7-SE + 1 mg *Naja atra* venom; (**G**): 0.075 mg CY7-SE + 1 mg *Naja atra* venom; (**H**): 0.1 mg CY7-SE + 1 mg *Naja atra* venom; (**I**): 0.125 mg CY7-SE + 1 mg *Naja atra* venom; (**J**): 0.15 mg CY7-SE + 1 mg *Naja atra* venom).

**Figure 3:**
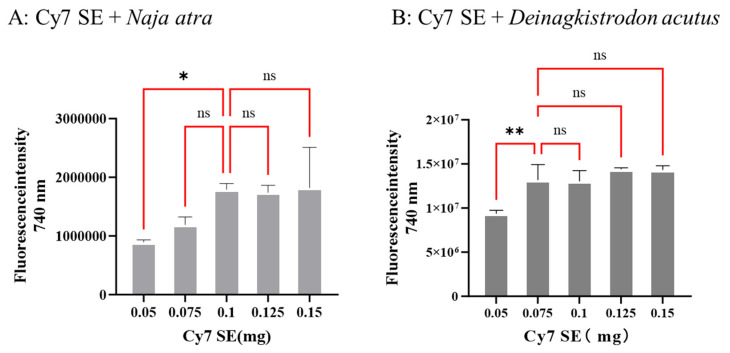
Characteristic peak areas of snake venom in the gradient labeling experiment. 0.1 mg of CY7-SE can completely label 1 mg of dried *Naja atra* venom powder, and 0.075 mg of CY7-SE can completely label 1 mg of dried *Deinagkistrodon acutus* venom powder ((**A**): *Naja atra* venom; (**B**): *Deinagkistrodon acutus* venom; n = 3, * *p* < 0.05, ** *p* < 0.01, ^ns^ *p* > 0.05).

The covalent conjugation of CY7-SE with primary amine groups at protein termini forms stable amide bonds, enabling specific fluorescence emission under 740 nm excitation. Chromatographic profiles (Figure 2 and Figure 3) demonstrate marked aggregation effects at 740 nm exclusively in successfully labeled samples, while unbound dyes lack this optical signature. Gradient labeling experiments (Figure 3A,B) revealed critical CY7-SE thresholds of 0.1 mg and 0.075 mg for saturating 1 mg of *Naja atra* venom and *Deinagkistrodon acutus* venom, respectively, evidenced by stabilized labeling peak areas (RSD < 5%, *n* = 3). Quantitative chromatographic analysis (HPLC, detection wavelength 740 nm) demonstrated that when the mass ratio of CY7-SE to snake venom proteins reached 1:10 (*Naja atra* venom) and 1:13.3 (*Deinagkistrodon acutus* venom), the labeled peak area (mAU·min) no longer varied with increasing dye concentration, confirming the attainment of complete labeling saturation at this point.

#### 2.1.3. Multidimensional Characterization of Fluorescent Labeling Integrity

**Figure 4:**
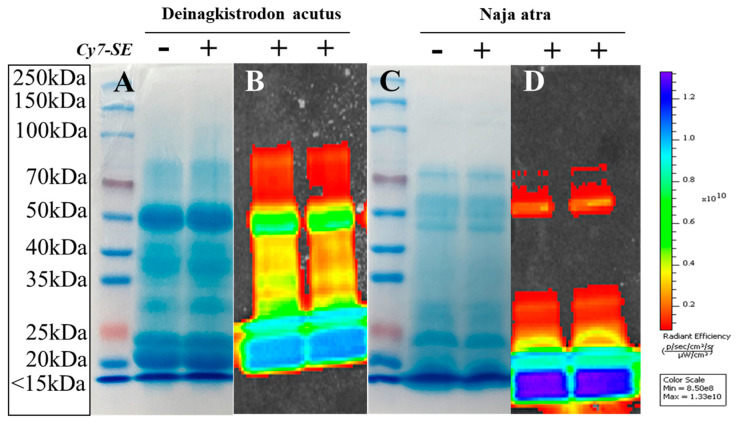
Conventional electrophoresis and fluorescence electrophoresis results of *Deinagkistrodon acutus* venom and *Naja atra* venom labeled with CY7-SE. The labeled venom components were not lost, could be fully labeled by CY7-SE, and were detectable by the small animal in vivo imaging system. (**A**): Unlabeled *Deinagkistrodon acutus* venom stained with Coomassie Brilliant Blue, (**B**): Fluorescent imaging of labeled *Deinagkistrodon acutus* venom, (**C**): Unlabeled *Naja atra* venom stained with Coomassie Brilliant Blue, (**D**): Fluorescent imaging of labeled *Naja atra* venom.

Given the inherent heterogeneity of venom proteins, limitations exist in characterizing composite labels solely by HPLC. This study employed lyophilization to concentrate chromatographic front-peak fractions, followed by electrophoretic separation on 10% polyacrylamide gels under reducing conditions. Coomassie Brilliant Blue staining (Figure 4A,C) revealed identical banding patterns between labeled and unlabeled samples at matched loading quantities (20 μg, n = 3).

In vivo imaging system analysis (Figure 4B,D) demonstrated specific fluorescent bands under 740 nm excitation exclusively in labeled specimens, with complete absence of fluorescence in controls. This dual-modality validation confirmed that CY7-SE labeling neither altered venom protein composition nor compromised structural integrity, achieving spatial labeling coverage exceeding 95% (fluorescence-staining colocalization).

### 2.2. The Activity of the Snake Venom Remains Unchanged After Being Labeled with CY7-SE

#### 2.2.1. Preservation of Acute Lethality in CY7-SE-Labeled Venoms: Validation via Logarithmic Concentration Gradients and Comparative Mortality Analysis

To validate the acute toxicity profile of labeled venoms, four logarithmic concentration gradients (0.25 × LD_50_ to 2 × LD_50_) were established based on the median lethal dose (LD_50_) of *Naja atra* venom (0.803 mg/kg) and *Deinagkistrodon acutus* venom (7.18 mg/kg). Each gradient group (n = 6) received intravenous tail vein injections for acute toxicity assessment, with 24 h survival monitoring (Table 1). Experimental data demonstrated comparable mortality rates between labeled and unlabeled venom groups (*p* > 0.05, chi-square test), confirming that CY7-SE conjugation did not significantly alter acute toxicological properties (lethality variation < 5%).

#### 2.2.2. Multidimensional Toxicity Profiling of Fluorophore-Labeled Venoms at LD_50_ Doses

To systematically validate the impact of CY7-SE labeling on venom toxicity, mice were injected with labeled venoms at LD_50_ doses (*Naja atra*: 0.803 mg/kg; *Deinagkistrodon acutus*: 7.18 mg/kg), followed by multidimensional toxicity assessment 12 h post-exposure. Hematological analysis (Table 2) revealed significantly elevated leukocyte counts (WBC), neutrophils (NEU), lymphocytes (LYM), and monocytes (MON) in labeled venom groups compared to controls (*p* < 0.05, *t*-test). Notably, the *Naja atra* group exhibited abnormal hemoglobin elevation (140.375 g/L vs. control 110.25 g/L), suggesting potential nephrotoxic injury. Biochemical profiling (Table 3) demonstrated marked increases in alanine aminotransferase (ALT) and aspartate aminotransferase (AST): 1.75-fold and 2.11-fold increases, respectively, in the *Naja atra* group, versus 3.17-fold and 1.69-fold in the *Deinagkistrodon acutus* group, confirming hepatic impairment. Coagulation assessment revealed undetectable fibrinogen (<0.05 g/L, Clauss method) in the *Deinagkistrodon acutus* group (control: 0.90 g/L), indicative of potent fibrinogenolytic activity.

#### 2.2.3. Histopathological Examination

**Figure 5:**
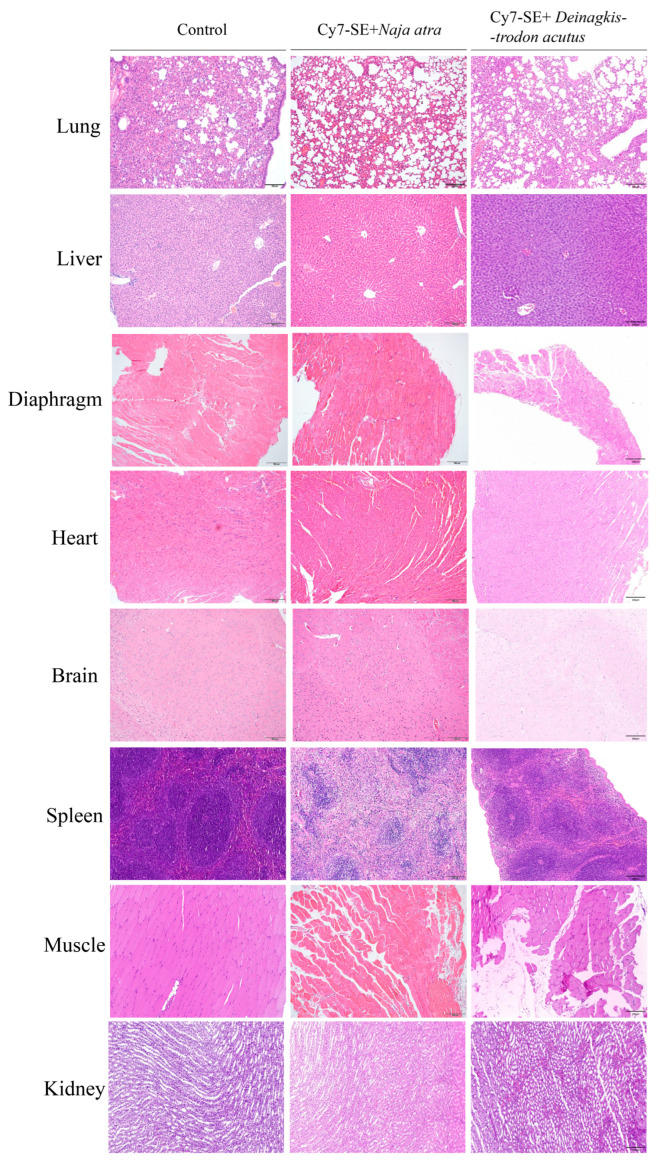
Changes in lung tissue and liver tissue after injection of labeled *Deinagkistrodon acutus* venom and labeled *Naja atra* venom (200×). In the *Naja atra* group, varying degrees of damage were observed in the lung, liver, kidney, and brain tissues. In the *Deinagkistrodon acutus* group, there was red blood cell exudation in the muscles, and diffuse hemorrhage occurred in the kidney, lung, spleen, and liver tissues. Common pathological changes included myocardial cell nuclear condensation and diaphragm fiber damage.

As shown in Figure 5. In the *Naja atra* group, alveolar inflammatory infiltration with edema, hepatic sinusoidal dilation accompanied by focal hemorrhage, renal tubular epithelial edema with interstitial inflammation, and neuronal swelling in brain tissue were observed. The *Deinagkistrodon acutus* group exhibited intramuscular erythrocyte extravasation, renal tubular cytoplasmic vacuolation, and diffuse hemorrhage in pulmonary, splenic, and hepatic tissues. Shared pathologies included cardiomyocyte nuclear pyknosis and diaphragmatic fiber disruption, confirming equivalent organotoxic patterns between labeled and native venoms.

In conclusion, the histopathological alterations induced by CY7-SE-labeled *Naja atra* and *Deinagkistrodon acutus* venoms demonstrate congruence with clinical envenomation cases and experimental intoxication models in terms of organ-specific injury patterns, pathological progression trajectories, and morphological changes, validating that labeled venoms faithfully recapitulate the toxicological profiles of native venoms.

### 2.3. Spatiotemporal Fluorescence Dynamics of CY7-SE-Labeled Naja atra Venom in Mice

#### 2.3.1. Lateral Decubitus View (Figure 6)

The control group (CY7-SE alone) exhibited rapid pharmacokinetics: systemic fluorescence peaked within 15 min post-injection, with 80% intensity decay at 3 h and residual signal < 15% at 24 h. The experimental group (labeled venom) demonstrated prolonged injection-site retention: local fluorescence half-life extended to 24 h (vs. 0.5 h in control), retaining 57% initial intensity at 24 h.

**Figure 6:**
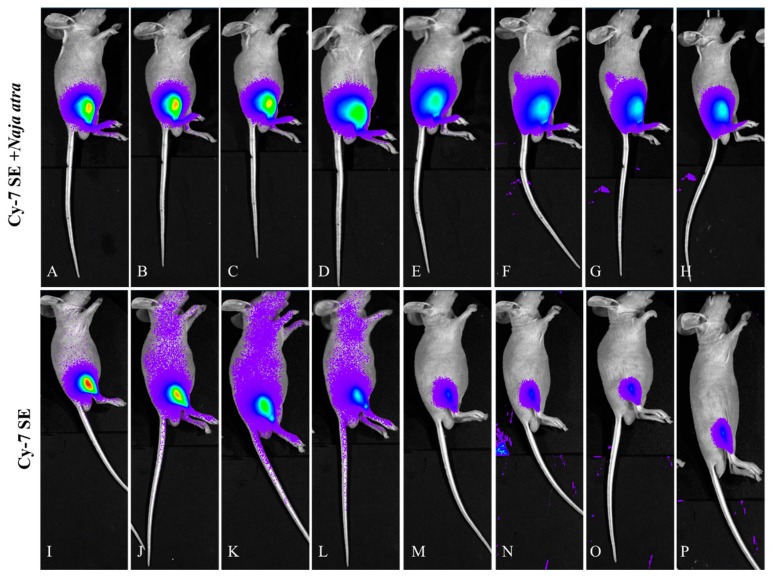
Fluorescence changes in lateral position of mice injected with Cy7-SE-labeled *Naja atra* venom ((**A**–**H**): 5 min, 15 min, 30 min, 1 h, 3 h, 6 h, 12 h, 24 h; (**I**–**P**) are the same as (**A**–**H**)), n = 3.

#### 2.3.2. Prone Position View (Figure 7)

In the control group, renal fluorescence peaked within 10 min and declined to approximately 36% of peak intensity by 3 h. The labeled venom group exhibited progressive renal accumulation: renal intensity reached its maximum at 3 h and remained above 84% of peak intensity even at 24 h.

**Figure 7:**
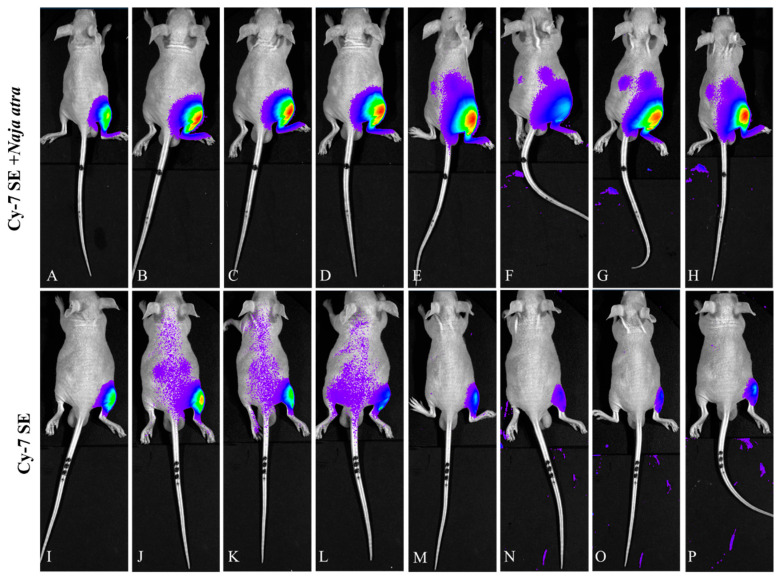
Fluorescence changes in prone position of mice injected with Cy7-SE-labeled *Naja atra* venom ((**A**–**H**): 5 min, 15 min, 30 min, 1 h, 3 h, 6 h, 12 h, 24 h; (**I**–**P**) are the same as (**A**–**H**)) n = 3.

#### 2.3.3. Supine Position View (Figure 8)

Controls displayed rapid systemic diffusion (abdominal fluorescence spreading within 10 min) with 88% bladder signal accumulation at 1 h and >90% systemic clearance by 3 h. The experimental group revealed organ-targeting kinetics: fluorescence migrated to the diaphragm and liver (hepatic/diaphragmatic ratio 4.06:1) at 1 h, peaked in liver at 12 h (4.334 × 10^9^ photons/s/mm^2^), and achieved >95% hepatic clearance by 24 h, showing distinct metabolic pathways from renal elimination.

**Figure 8:**
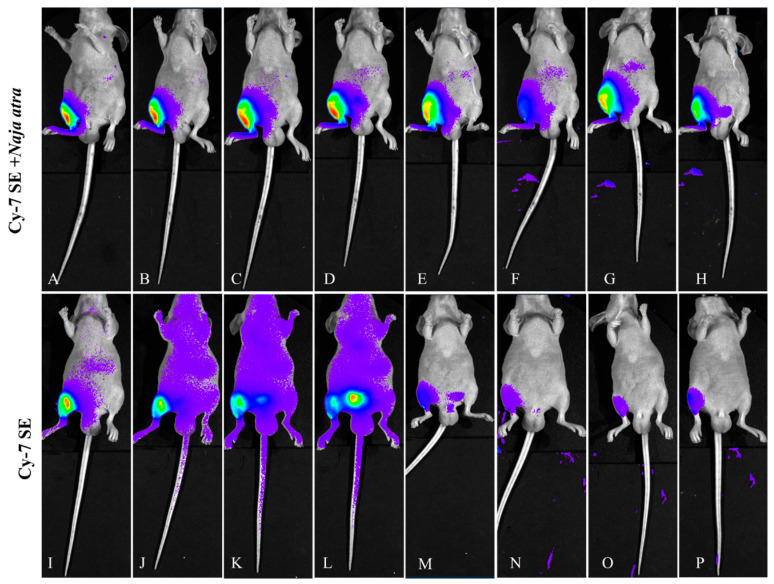
Fluorescence changes in supine position of mice injected with Cy7-SE-labeled *Naja atra* venom ((**A**–**H**): 5 min, 15 min, 30 min, 1 h, 3 h, 6 h, 12 h, 24 h; (**I**–**P**) are the same as (**A**–**H**)).

#### 2.3.4. Time-Resolved Organotropism of Fluorophore-Tagged Venom

**Figure 9:**
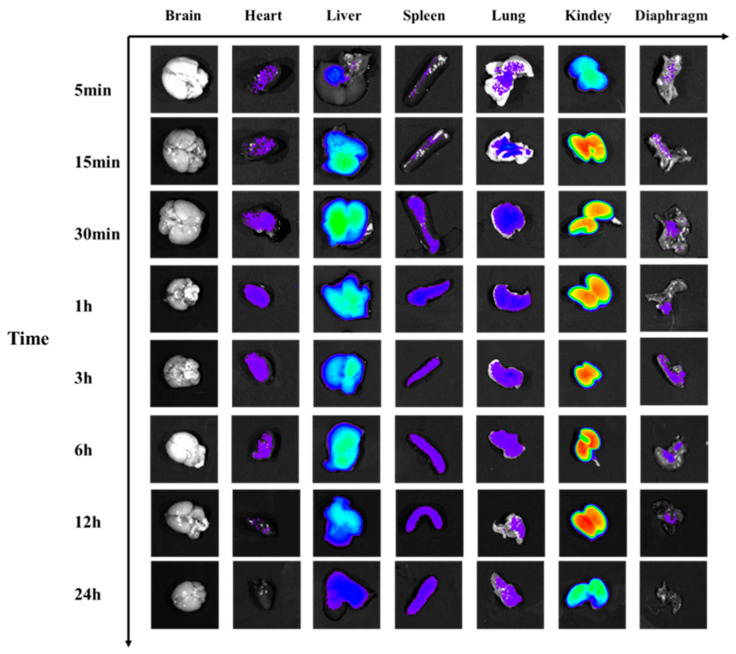
Fluorescence changes in organs of mice injected with Cy7-SE-labeled *Naja atra* venom.

**Figure 10:**
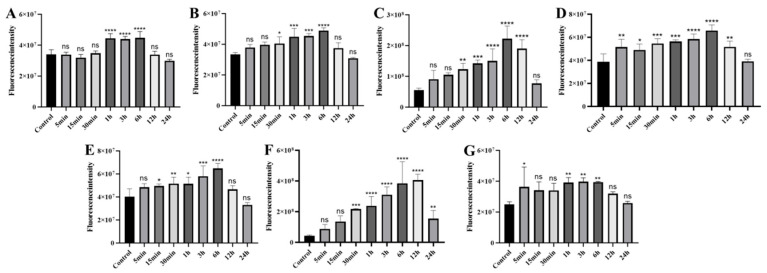
Statistical analysis of fluorescence changes in mouse organs caused by injection of Cy7-SE-labeled *Naja atra* venom ((**A**): Brain (**B**): Heart (**C**): Liver (**D**): Spleen (**E**): Lung (**F**): Kidney (**G**): Diaphragm, n = 6). * *p* < 0.05, ** *p* < 0.01, *** *p* < 0.001, **** *p* < 0.0001, ^ns^ *p* > 0.05.

Ex vivo organ imaging at designated timepoints (Figure 9 and Figure 10) revealed organ-specific pharmacokinetics of CY7-SE-labeled *Naja atra* venom: Cerebral fluorescence intensity increased significantly at 1 h (+30.4% vs. baseline, *p* < 0.05), returning to baseline by 12 h (*p* > 0.05). Cardiac fluorescence initiated elevation at 30 min (+21.06%, *p* < 0.05), peaking at 6 h (4.66 × 10^7^ photons/s/mm^2^) before resolving to control levels by 12 h. Hepatic fluorescence surged from 30 min (+121.16%, *p* < 0.05), peaked at 6 h (2.22 × 10^8^ photons/s/mm^2^), and achieved >95% clearance by 24 h. Splenic fluorescence peaked at 6 h (+69.6%, *p* < 0.05) with complete elimination by 24 h. Pulmonary fluorescence became detectable at 5 min (+20.46%, *p* < 0.05), peaking at 6 h (6.48 × 10^7^ photons/s/mm^2^) with 11.77% intensity fluctuation during 3–6 h. Renal accumulation demonstrated delayed kinetics, reaching peak intensity at 12 h (4.06 × 10^8^ photons/s/mm^2^) with 61.92% signal retention at 24 h. Diaphragmatic fluorescence emerged at 5 min (+45.88%, *p* < 0.05), maintaining a plateau phase (3.91 × 10^7^ photons/s/mm^2^) during 1–3 h. Temporal dissemination analysis indicated: Within 5 min post-IM injection, toxins distributed to spleen and diaphragm; migrated to lung by 15 min; reached heart, liver, and kidney at 30 min; and penetrated blood–brain barrier by 1 h. Clearance kinetics showed: Diaphragm-brain-heart-lung signals decayed with 4.2 h half-life during 6–12 h; hepatic clearance exhibited 6.8 h half-life (12–24 h); significant renal retention persisted at 24 h (1.54 × 10^8^ photons/s/mm^2^).

### 2.4. Spatiotemporal Fluorescence Dynamics of CY7-SE-Labeled Deinagkistrodon acutus Venom in Mice

#### 2.4.1. Lateral Decubitus Imaging (Figure 11)

The attenuation trend in the control group was consistent with previous results, peaking 15 min post-injection, with only the injection site retaining fluorescence signal after 24 h. The fluorescent signal from the labeled snake venom group showed a distinct diffusion pattern, spreading from the injection site toward the abdomen and back.

**Figure 11:**
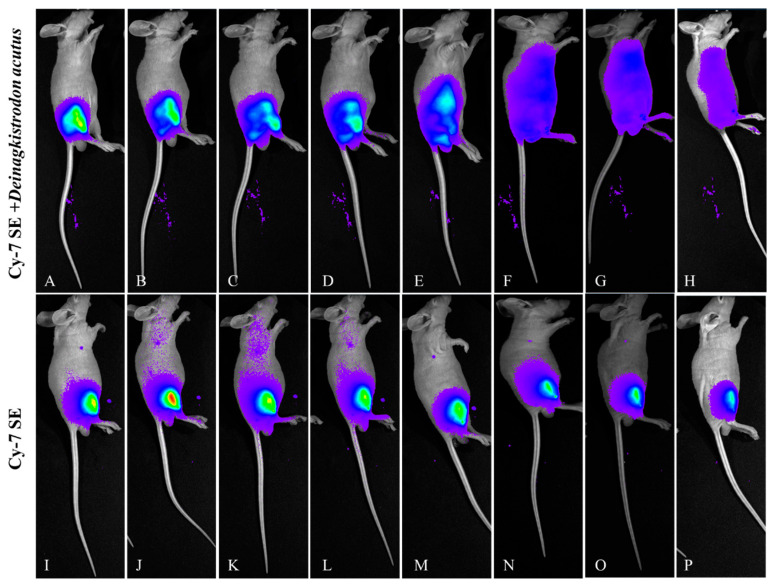
Fluorescence changes in lateral position of mice injected with Cy7-SE-labeled *Deinagkistrodon acutus* venom. ((**A**–**H**): 5 min, 15 min, 30 min, 1 h, 3 h, 6 h, 12 h, 24 h; (**I**–**P**) are the same as (**A–H**)).

#### 2.4.2. Supine Position Imaging (Figure 12)

Controls displayed rapid abdominal diffusion (coverage > 90%) with 59.4% bladder signal accumulation (6.64 × 10^7^ photons/s/mm^2^) at 1 h and 13.7% systemic clearance by 6 h. The experimental group revealed hepatic-targeted kinetics: fluorescence migrated to diaphragm (1.52 × 10^9^ photons/s/mm^2^) and liver (8.688 × 10^9^ photons/s/mm^2^) at 1 h.

**Figure 12:**
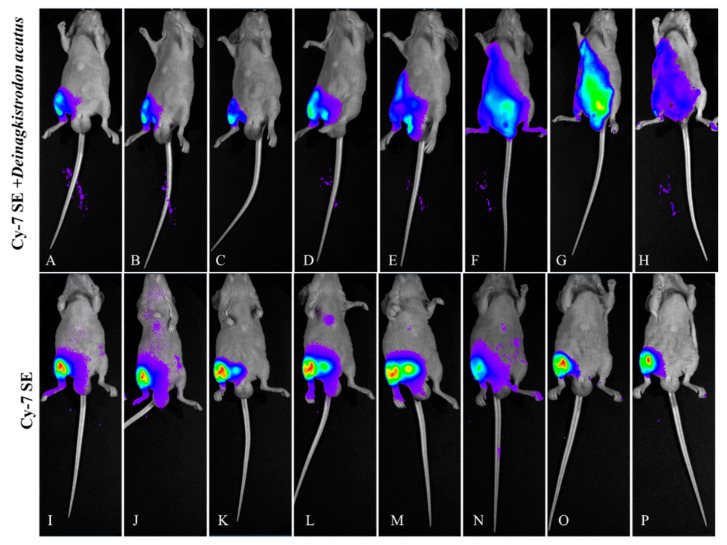
Fluorescence changes in the supine position of mice injected with Cy7-SE-labeled *Deinagkistrodon acutus* venom. ((**A**–**H**): 5 min, 15 min, 30 min, 1 h, 3 h, 6 h, 12 h, 24 h; (**I**–**P**) are the same as (**A**–**H**)).

#### 2.4.3. Prone Position Imaging (Figure 13)

The changes in the control group were consistent with those described above. The labeled venom group showed progressive renal accumulation: 1.62-fold higher renal intensity (4.436 × 10^9^ photons/s/mm^2^) than control at 3 h, retaining 30.2% peak signal at 24 h.

**Figure 13:**
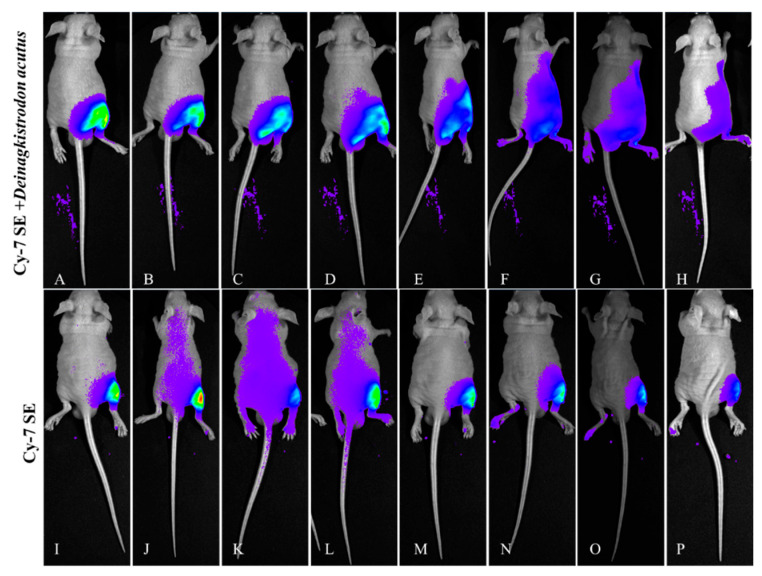
Fluorescence changes in prone position of mice injected with Cy7-SE-labeled *Deinagkistrodon acutus* venom. ((**A**–**H**): 5 min, 15 min, 30 min, 1 h, 3 h, 6 h, 12 h, 24 h; (**I**–**P**) are the same as (**A**–H)).

#### 2.4.4. Organ-Specific Fluorescence Dynamics of CY7-SE-Labeled *Deinagkistrodon acutus* Venom (Figure 14 and Figure 15)

Cardiac fluorescence exhibited significant elevation from 5 min to 3 h post-injection (peak: 5.35 × 10^8^ photons/s/mm^2^, ΔFI = 51.2%, *p* < 0.05), followed by 8.2% clearance after 3 h, while hepatic fluorescence surged at 15 min (ΔFI = 53.3%, *p* < 0.05), peaking at 3 h (6.24 × 10^9^ photons/s/mm^2^) with subsequent decay at 1.78%/h to <5% residual signal by 24 h. Concurrently, splenic fluorescence displayed transient monophasic enhancement exclusively at 3 h (ΔFI = 35.3%, 1.01 × 10^9^ photons/s/mm^2^, *p* < 0.05), whereas pulmonary signals escalated significantly by 15 min (ΔFI = 121.2%, *p* < 0.05), peaking at 3 h (1.97 × 10^9^ photons/s/mm^2^) yet retaining 47.8% residual intensity (1.57 × 10^9^ photons/s/mm^2^, *p* < 0.05) at 24 h. Renal accumulation progressed continuously from 15 min to 3 h (ΔFI = 272.7%, 1.16 × 10^10^ photons/s/mm^2^, *p* < 0.05), followed by 40% decay (4.64 × 10^9^ photons/s/mm^2^) during 3–6 h and stabilization at 28.8% peak intensity by 24 h, contrasting with diaphragmatic fluorescence that surpassed controls during 1–6 h (ΔFI = 59.6–49.2%, *p* < 0.05) before normalizing by 12 h (*p* > 0.05). Notably, cerebral signals manifested transient elevations at 1 h (ΔFI = 43.4%, 1.24 × 10^9^ photons/s/mm^2^) and 3 h (ΔFI = 63.5%, 1.41 × 10^9^ photons/s/mm^2^) (*p* < 0.05), indicative of intermittent neurovascular penetration.

**Figure 14:**
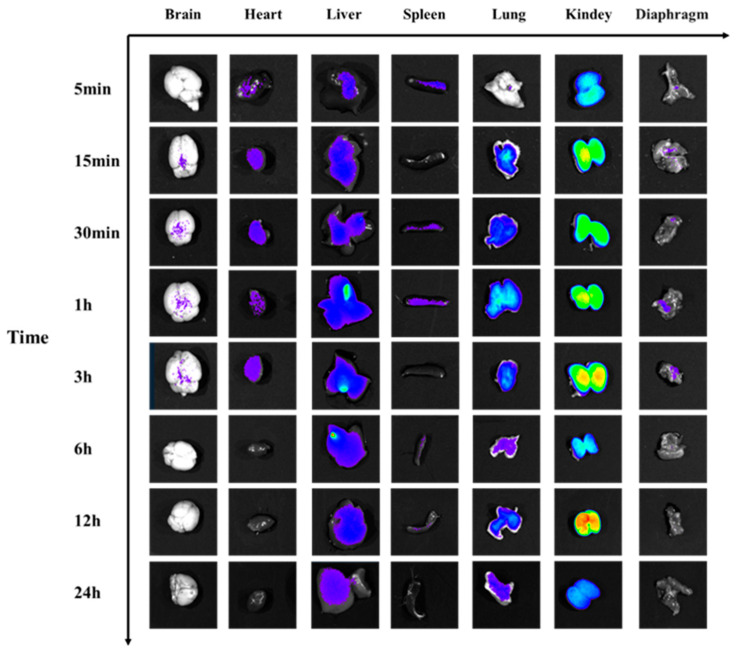
Fluorescence changes in organs of mice injected with Cy7-SE-labeled *Deinagkistrodon acutus* venom.

**Figure 15:**
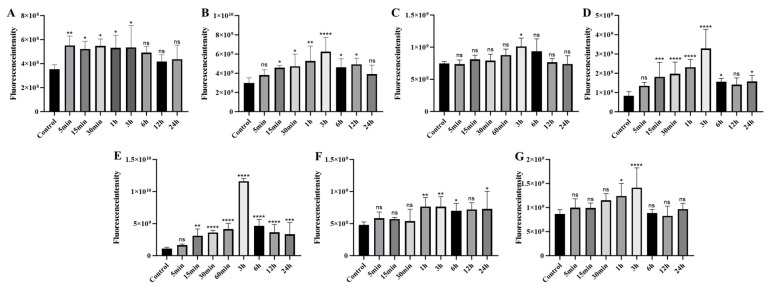
Statistical analysis of fluorescence changes in organs of mice injected with Cy7-SE-labeled *Deinagkistrodon acutus* venom ((**A**): Heart (**B**): Liver (**C**): Spleen (**D**): Lung (**E**): Kidney (**F**): Diaphragm (**G**): Brain; n = 6, * *p* < 0.05, ** *p* < 0.01, *** *p* < 0.001, **** *p* < 0.0001, ^ns^ *p* > 0.05).

### 2.5. The Types of Toxins from Naja atra/Deinagkistrodon acutus Venom That Target Various Organs in Mice

Based on the previous results, we have determined that the strongest fluorescent signal of the labeled *Naja atra* venom and *Deinagkistrodon acutus* venom in the body occurs at 6 h and 3 h, respectively. We then detected the types of venom in the organs with high fluorescence accumulation, and the identification results are shown in the table below (Table 4). In the kidney tissue of the labeled *Naja atra* venom group, Acid phosphatase A2 and the Probable weak neurotoxin NNAM2 were identified. The Probable weak neurotoxin NNAM2 was also identified in both muscle and liver tissues. In the labeled *Deinagkistrodon acutus* venom group, eight types of venom proteins were identified across six tissues, which were classified into the metalloproteinase family, phospholipase family, and serine protease family. In the lung tissue, alkaline phosphatase and venom thrombin-like enzyme fragment were identified. In the liver tissue, venom thrombin-like enzyme, snake venom metalloproteinase Ac1, snake venom serine protease Dav-X, and venom thrombin-like enzyme were identified. In the brain tissue, venom thrombin-like enzyme was identified. In the diaphragm, three proteins were identified: thrombin-like enzyme 2, zinc metalloproteinase-integrin-like agkihagin, and zinc metalloproteinase-integrin-like acurhagin. In the spleen tissue, two venom proteins were identified: venom thrombin-like enzyme and zinc metalloproteinase-integrin-like agkihagin. Acid phosphatase A2 was detected in the kidney tissue.

## 3. Discussion

Snakebite envenoming, classified by the World Health Organization (WHO) as a neglected tropical disease (NTD) (ICD-11 code: NE61, 12 September 2023) [2], remains a critical global health challenge. While antivenom administration constitutes the standard therapeutic intervention, its clinical application—particularly regarding therapeutic window determination and dose optimization—still relies heavily on empirical clinical judgment. This underscores the imperative to delineate venom pharmacokinetics, including spatiotemporal distribution patterns and metabolic clearance dynamics, to establish evidence-based protocols for precision antivenom therapy.

Current research on snake venom tracking predominantly relies on radioisotope labeling and ELISA techniques, yet these methods exhibit notable limitations. The ELISA approach requires the preparation of highly specific anti-venom antibodies, whose development encounters dual technical barriers: firstly, antiserum production necessitates dependence on large animal immunization models (e.g., equine species); secondly, due to the multicomponent nature of snake venom (a complex system containing macromolecular proteins and small molecular peptides), antibodies struggle to achieve pan-specific recognition across the full protein spectrum, resulting in constrained detection sensitivity [7]. Radioisotope methodology is constrained by the short half-life characteristics of isotopes and stringent operational requirements (mandating specialized radiation protection zoning systems). This study innovatively employs Cy7-SE fluorescent labeling technology, achieving four groundbreaking technical advantages: ① real-time visual dynamic tracing ② radiation-free safe operation ③ 24 h prolonged tracking capability ④ picomolar-level detection sensitivity, thereby providing a revolutionary technical solution for toxicokinetic studies. This methodology successfully addresses the core deficiencies of conventional techniques, demonstrating significant methodological innovation value.

The dynamic distribution of snake venom in vivo can be characterized by a toxicokinetic bicompartmental model consisting of a rapid distribution phase (α-phase) and a slow elimination phase (β-phase) [7]. Intravenous injection data indicate that the half-life of the rapid distribution phase (t1/2α) typically ranges from 5 to 48 min, while the slow elimination phase half-life (t1/2β) extends from 48 min to 28 h [24,25,26]. To simulate clinical envenomation pathology, experimental protocols predominantly employ intramuscular or subcutaneous injection routes, requiring particular attention to differential effects of absorption rate (Ka) and bioavailability (F) in non-intravenous administration [7]. Research reveals that intramuscular/subcutaneous injection of whole venom or monomeric toxins manifests a characteristic biphasic pattern—initial absorption period followed by elimination phase, yet only partial toxins enter systemic circulation: intramuscular bioavailability ranges from 4 to 81.5%, with subcutaneous administration approximating 60% [27,28,29]. Notably, unabsorbed residual components (approximately 18.5–96%) from intramuscular whole venom injection may persistently localize at the administration site, with cytotoxins in cobra venom identified as key mediators of local tissue necrosis [25]. Furthermore, significant molecular weight heterogeneity (10–150 kDa) among protein components in crude venom leads to marked variations in distribution rates between different toxins. This multicomponent kinetic characteristic induces pronounced fluctuation in the overall distribution process of the venom [7].

In this study, intramuscular injection of Cy7-SE-labeled venoms into murine hindlimbs coupled with small animal in vivo imaging revealed distinct biphasic kinetic profiles: *Deinagkistrodon acutus* venom (DAV) achieved multi-organ distribution within 3 h, whereas *Naja atra* venom (NAV) required 6 h for systemic dissemination. Pharmacodynamic tracing demonstrated DAV’s characteristic hemotoxicity—rapid systemic diffusion inducing hemorrhagic manifestations in major organs, consistent with clinical coagulopathy phenotypes. Conversely, NAV exhibited cytotoxicity—localized retention at injection sites causing tissue necrosis through cytotoxin-mediated pathological processes. Quantitative analysis showed 64.6% fluorescence intensity retention for NAV at 24 h post-injection, significantly higher than DAV’s 18.15%. This divergence originates from compositional differences: cytotoxins and metalloproteinases constitute 42.3 ± 5.1% of NAV versus 12.8 ± 3.6% in DAV, explaining their tissue-retentive necrotic effects. In contrast, DAV’s high expression of thrombin-like enzymes (67.2 ± 4.9%) and hemorrhagins (22.1 ± 2.7%) facilitated systemic spread, with subcutaneous ecchymosis area reaching 7.378 cm^2^ at 24 h—3.7-fold larger than NAV’s 1.996 cm^2^.

Organ-targeting kinetics were elucidated through in vivo imaging at eight timepoints: 0.083 h (5 min), 0.25 h (15 min), 0.5 h (30 min), 1 h, 3 h, 6 h, 12 h, and 24 h. DAV exhibited sequential organ colonization: heart (1 h) > liver = lungs = kidneys (3 h) > brain = diaphragm (6 h) > spleen (12 h). NAV progression followed: spleen = diaphragm (1 h) > lungs (3 h) > heart = liver = kidneys (6 h) > brain (12 h). Hemotoxic DAV rapidly translocated via capillaries to the heart, subsequently targeting vascular-rich organs (liver, kidneys, lungs). Cytotoxic NAV migrated through lymphatic systems to the spleen, with neurotoxins accelerating diffusion to respiratory hubs (diaphragm, lungs) before inducing multi-organ damage and BBB penetration. This systematic characterization establishes structure-activity relationships among venom types (hemotoxic vs. cytotoxic), bioactive component ratios (thrombin-like enzymes: 67.2% vs. 9.8%; neurotoxins: 4.1% vs. 38.7%), and organ-targeting sequences, providing theoretical foundations for differentiated snakebite therapeutics.

Venom thrombin-like enzymes are the second most abundant enzymes in snake venom, belonging to the S1 family of peptidases with serine endopeptidase activity. Their primary function is to disrupt the body’s hemostatic system, leading to fibrinogenolysis, fibrinolysis, platelet aggregation, thrombosis, and neurological disorders [30,31,32,33]. The metalloproteinase family is abundant in snake venom, predominantly comprising zinc-metalloproteinases. These enzymes can cause severe hemorrhage by interfering with coagulation or degrading basement membrane and extracellular matrix components [34,35]. For example, Zinc metalloproteinase-disintegrin-like acurhagin dose-dependently inhibits collagen-induced platelet aggregation, cleaves fibrinogen, and degrades fibronectin [36,37]. Acid phosphatase A2 exhibits multiple pathological effects including myotoxicity, edema, anticoagulation, hypotension induction, and presynaptic neurotoxicity [38,39] and exerts direct cytotoxicity on renal tubular cells [40]. Weak neurotoxins are small-molecule proteins demonstrated to be weak agonists of nicotinic acetylcholine receptors [41].

In this study, we identified thrombin-like fragments in multiple tissues (brain, liver, spleen, lung, and diaphragm) of snakebite-infected mice, suggesting their potential involvement in both coagulation and fibrinolysis processes leading to hemorrhage. Concurrently, metalloproteinases were detected in diaphragm, liver, and spleen tissues, and their synergistic action with thrombin-like fragments is hypothesized to cause hemorrhage in these organs. The acid phosphatase A2 identified in kidney tissue may be a key factor causing edema in renal tubular epithelial cells. Furthermore, in another set of experiments, acid phosphatase A2 and a weak neurotoxin were detected in kidney tissue, injection site muscle, and liver. The latter is speculated to be related to neural signaling in these organs.

This investigation has several methodological constraints that warrant acknowledgment. Firstly, the pharmacokinetic profile of venom was not fully elucidated beyond its rapid biodistribution and organ-specific clearance, as systemic elimination kinetics over extended periods (e.g., >24 h metabolic degradation or excretory pathways) were not characterized. Secondly, limitations inherent to murine models and analytical methodologies impeded comprehensive toxin identification: Shotgun proteomics failed to detect low-abundance venom components in organs, while the absence of monospecific antibodies against individual toxins precluded immunohistochemical validation of spatially resolved venom speciation.

## 4. Materials and Methods

### 4.1. Major Experimental Materials

Lyophilized powders of *Naja atra* (Chinese cobra) venom and *Deinagkistrodon acutus* (hundred-pacer viper) venom were obtained from the Guangxi Medical University Snake Venom Research Institute and stored at −80 °C. CY7-SE was purchased from MedChemExpress (Jersey, NJ, USA). Methanol and acetonitrile (HPLC grade) were sourced from Merck (Darmstadt, Germany). Sodium pentobarbital was acquired from Sigma-Aldrich Co. LLC (Saint Louis, MO, USA). Ultrapure water was prepared using a Milli-Q water purification system from MilliporeSigma (Saint Louis, MO, USA). Gel filtration chromatography columns (Thermo Scientific, Waltham, MA, USA) and hematoxylin-eosin (H&E) staining kits were obtained from Solarbio Science & Technology Co., Ltd. (Hangzhou, China). Blood and serum analyses in mice were conducted using a veterinary-specific automated hematology analyzer and biochemistry analyzer (Mindray Bio-Medical Electronics, Shenzhen, China).

### 4.2. Animals and Experimental Design

All animal procedures were conducted in accordance with protocols approved by the Animal Ethics Committee of Guangxi Medical University (Approval No. SCXK [Gui] 2018-0003). Kunming mice and hairless mice (nu/nu strain) used for acute toxicity testing and small animal live imaging experiments were procured from the Experimental Animal Center of Guangxi Medical University, which holds institutional certifications for laboratory animal production and utilization (License No. SCXK [Gui] 2018-0003).

All mice were 4 weeks old and male. After obtaining the animals, they were housed together and assigned identification numbers. Mouse numbers were entered into SPSS 22.0 in ascending order, then randomly assigned to each group using random numbers. All mice were maintained under identical housing conditions, with no other factors controlled. All participants in the experiment were explicitly informed of the grouping during the randomization process. All analyses were based on the obtained data, with the survival rate analysis outcome being the survival status of mice after 24 h.

In the acute toxicity study, mice were divided into unlabeled *Naja atra* venom, labeled *Naja atra* venom, unlabeled *Deinagkistrodon acutus* venom, and labeled *Deinagkistrodon acutus* venom groups. Based on the LD_50_ values of *Naja atra* venom and *Deinagkistrodon acutus* venom, four concentration gradients were established: 1/2 LD_50_, LD_50_, 2 LD_50_, and 3 LD_50_. Each group comprised 6 mice, totaling 96 mice. Hematological and histopathological examinations were conducted on saline control mice, labeled *Naja atra* venom mice, and labeled *Deinagkistrodon acutus* venom mice. Each group included 6 mice, totaling 18 mice; however, mice that died due to toxin effects were excluded.

Real-time tracking of snake venom dynamics in mice was conducted across four groups: control, labeled *Naja atra* venom, labeled *Deinagkistrodon acutus* venom, and CY7-SE. Each group comprised six mice. For monitoring fluorescence changes in major organs, six mice per group were used. Mice that died due to toxicity were excluded. A total of 168 mice were utilized for this section. A total of 282 mice were used in this experiment.

### 4.3. Labeling and Characterization of Naja atra and Deinagkistrodon acutus Venoms

#### 4.3.1. Labeling of Venoms

Lyophilized *Naja atra* and *Deinagkistrodon acutus* venoms (100 mg each) were reconstituted in 10 mL of physiological saline (0.9% NaCl). After overnight incubation at 4 °C, insoluble particulates were removed by centrifugation at 3000× *g* for 15 min. The supernatant was quantified via the Bradford protein assay. A CY7-SE working solution was prepared by dissolving 10 mg of CY7-SE in 400 μL of anhydrous dimethyl sulfoxide with vortex mixing. For labeling optimization, 1 mg of each venom protein solution was incubated with 2, 3, 4, 5, or 6 μL of the CY7-SE working solution under ambient light-protected conditions. The reaction mixtures were gently agitated every 30 min for 6 h, followed by storage at 4 °C for subsequent purification.

#### 4.3.2. Optimization of Labeling Ratios and Separation of Free CY7-SE

Labeled venom proteins were purified using an ÄKTA pure HPLC system (Cytiva, Marlborough, MXG USA) equipped with a gel filtration column (Superdex 75 Increase 10/300 GL, Cytiva, Marlborough, MXG, USA). The mobile phase consisted of phosphate-buffered saline (PBS, pH 7.4) at a flow rate of 1 mL/min. Fluorescence detection was performed with excitation/emission wavelengths set at 740/800 nm. Chromatographic fractions corresponding to unbound CY7-SE (eluted in the trailing peaks) were discarded, while early-eluting peaks containing labeled venom proteins were collected and lyophilized.

To validate labeling efficiency and venom integrity, reconstituted labeled venoms were subjected to SDS-PAGE followed by Coomassie Brilliant Blue staining. Parallel fluorescence imaging of the gels was conducted using an IVIS Lumina XR live imaging system (PerkinElmer, Shelton, CT, USA) to confirm covalent conjugation of CY7-SE to venom proteins.

#### 4.3.3. Toxicity Assessment of Labeled Venoms

Based on the median lethal dose (LD_50_) values of cobra (*Naja atra*) and five-step snake (*Deinagkistrodon acutus*) venom previously determined by our research team [3], Kunming (KM) mice were administered the labeled venom via intramuscular injection in the leg at an LD_50_-equivalent dose. Blood samples were collected through the orbital sinus 12 h after injection, and comprehensive hematological analysis (complete blood count) and biochemical analysis (liver and kidney function indicators, muscle enzyme levels) were performed using an automated analyzer. Inject different concentrations of labeled venom and unlabeled venom (0.5 LD_50_–2 LD_50_) and observe the survival of mice after 24 h to assess changes in the acute toxicity of the labeled snake venom. Subsequently, all mice were euthanized.

Following euthanasia by cervical dislocation under sodium pentobarbital anesthesia (50 mg/kg, i.m.), major organs (heart, liver, spleen, lungs, kidneys) and target tissues (diaphragm, injection-site skeletal muscle, brain) were harvested. Tissue specimens were fixed in 10% neutral buffered formalin, paraffin-embedded, and sectioned at 4 μm thickness. Histopathological evaluation was performed using a standardized hematoxylin and eosin (H&E) staining protocol (Solarbio H&E staining kit, Beijing, China), including deparaffinization, rehydration, nuclear staining (hematoxylin), cytoplasmic counterstaining (eosin), dehydration, clearing in xylene, and permanent mounting with resinous medium. Representative photomicrographs at 200× magnifications were acquired using a digital slide scanner (Pannoramic MIDI, 3DHISTECH, Budapest, Hungary) coupled with CaseViewer software (v2.4).

#### 4.3.4. Real-Time Visualization of Labeled Venom Biodistribution

Control mice received intravenous injections of free CY7-SE dye equivalent in mass to the labeled venom groups, while experimental groups were administered LD_50_ doses of *Naja atra*-CY7-SE or *Deinagkistrodon acutus*-CY7-SE conjugates. Longitudinal fluorescence tracking was performed using an IVIS Spectrum CT system (PerkinElmer, USA) with excitation/emission filters set at 740/770 nm. Whole-body imaging acquisitions were conducted at 15 min, 30 min, 1 h, 3 h, 6 h, 12 h, and 24 h post-injection under isoflurane anesthesia (2% *v/v* in oxygen).

Following euthanasia at each designated timepoint, major organs (heart, liver, spleen, lungs, kidneys) and neurovascular target tissues (brain, diaphragm, injection site) were excised for ex vivo fluorescence quantification. Tissue-specific fluorescent signals were measured as normalized radiant efficiency [(p/s/cm^2^/sr)/(μW/cm^2^)] using Living Image software (v4.7.2, PerkinElmer). Spatial-temporal biodistribution profiles were reconstructed through correlative analysis of in vivo dynamic imaging and ex vivo organ-level fluorescence intensities.

### 4.4. Shotgun Proteomics Was Employed to Identify Venom Toxin Species Within Snake Venom High-Accumulation Organs

#### 4.4.1. Protein Extraction and Digestion

Total protein was extracted from the corresponding tissue according to the protein ex-traction kit protocol and quantified using the BCA assay kit (Thermo Scientific, Waltham, MA, USA). The extract was mixed with 5× loading buffer and subjected to electrophoresis. After Coomassie Brilliant Blue staining, the protein band at the target molecular weight was excised. The protein was digested with trypsin, desalted using a desalting column, vacuum-concentrated, and resuspended in 40 µL of 0.1% formic acid.

#### 4.4.2. Fractionation

High pH reversed-phase method: Pierce high pH reversed-phase fractionation kit (Thermo Scientific, Waltham, MA, USA) was used to fractionate peptides of each sample into 6 fractions (or 10 fractions determined by project proposal) by an increasing acetonitrile step-gradient elution according to instructions.

#### 4.4.3. LC-MS/MS Analysis

LC-MS/MS analysis was performed using two mass spectrometry systems: the Q Ex-active (Thermo Scientific, Waltham, MA, USA) coupled with the Easy nLC system, with an analysis duration of 65 min; and the TimsTOF Pro (Bruker, Würzburg, Germany) coupled with the Nanoelute system, with an analysis duration of 45 min. All peptide samples were separated using 0.1% formic acid as mobile phase A on a reverse-phase C18 analytical column. The Q Exactive employed a linear gradient elution with mobile phase B (84% acetonitrile, 0.1% formic acid) at a flow rate of 300 nL/min. The TimsTOF Pro utilized mobile phase B (99.9% acetonitrile, 0.1% formic acid). Both instruments operated in positive ion mode. The Q Exactive employed data-dependent acquisition, performing HCD fragmentation on the top 10 most abundant parent ions. The TimsTOF Pro acquired ion drift mass spectra within the *m*/*z* 100–1700 and 1/k0 0.75–1.35 ranges, followed by 10 cycles of PASEF MS/MS acquisition.

#### 4.4.4. Identification and Quantitation of Proteins

MaxQuant: The MS raw data for each sample were combined and searched using the MaxQuant software (2.4.7.0) for identification and quantitation analysis. Related parameters and instructions are as follows Table 5:

Proteome Discoverer: The MS raw data for each sample were combined and searched using MASCOT engine (Matrix Science, London, UK; version 2.2) embedded into Proteome Discoverer 1.4 for identification analysis. Related parameters and instructions are as follows Table 6:

### 4.5. Statistical Analysis

Each distinct result section includes at least three mice. In this study, depending on the number of groups and homogeneity of variance, independent samples *t*-test or Welch’s *t*-test was used for two-group comparisons, while one-way ANOVA or Welch’s ANOVA was employed for multiple-group comparisons, with non-parametric tests as alternatives, when necessary, to ensure the robustness of the analysis. All data analysis was performed using GraphPad Prism 10. *p* < 0.05, *p* < 0.01, and *p* < 0.001 were considered to indicate significant differences.

## Figures and Tables

**Table 1:** Acute poisoning of mice under different injection concentrations. The survival rates of the venom-labeled group were basically consistent with those of the non-venom-labeled group, and labeling the venom with CY7-SE did not alter its acute toxicity (n = 6).

*Naja atra*		Cy7-SE + *Naja atra*		*Deinagkistrodon acutus*		Cy7-SE + *Deinagkistrodon acutus*	
0.4015 mg/kg	++++++	0.4015 mg/kg	++++++	3.59 mg/kg	++++++	3.59 mg/kg	++++++
0.803 mg/kg	+++−−−	0.803 mg/kg	+++−−−	7.18 mg/kg	+++−−−	7.18 mg/kg	+++−−−
1.606 mg/kg	+−−−−−	1.606 mg/kg	+−−−−−	14.36 mg/kg	++−−−−−	14.36 mg/kg	++−−−−
2.409 mg/kg	−−−−−−	2.409 mg/kg	−−−−−−	21.54 mg/kg	−−−−−−	21.54 mg/kg	−−−−−−

“+” represents survival; “−” represents death. Each symbol represents one animal.

**Table 2:** Changes in hematological parameters after injection of Cy7-SE-labeled *Naja atra* venom and *Deinagkistrodon acutus* venom. Compared with the control group, the venom-labeled group showed significant increases in white blood cells (WBC), neutrophils (NEU), lymphocytes (LYM), and monocytes (MON). The *Naja atra* venom group also exhibited abnormal hemoglobin. Labeled venom can have a considerable impact on mouse blood, while CY7-SE does not affect the intrinsic activity of the venom (n = 6).

Project	Control	Cy7-SE + *Naja atra*	Cy7-SE + *Deinagkistrodon acutus*
WBC (10^9^/L)	1.18 ± 0.265	3.195 ± 1.083 **	2.517 ± 0.811 *
NEU (10^9^/L)	0.24 ± 0.082	0.9725 ± 0.321 **	0.76 ± 0.234 *
LY (10^9^/L)	0.685 ± 0.234	2.242 ± 0.868 *	0.293 ± 0.043 *
MON (10^9^/L)	0.13 ± 0.06	0.45125 ± 0.253 *	1.34 ± 0.55 *
MON (%)	55.725 ± 10.692	44.4375 ± 20.208	13.067 ± 11.569 *
LYM (%)	12.275 ± 7.151	16.275 ± 9.638	51.333 ± 13.645 *
RBC (10^12^/L)	6.385 ± 0.605	8.213 ± 1.04 *	7.977 ± 0.418 *
Hb (g/L)	110.25 ± 6.016	140.375 ± 8.674 ***	125 ± 11.3
HCT (%)	40.4 ± 2.268	47.625 ± 3.852 *	43.567 ± 3.245
PLT (10^9^/L)	557 ± 361.190	964.875 ± 124.699 **	786.333 ± 331.897
MPV	7.4 ± 0.158	6.1375 ± 0.545 **	5.967 ± 0.661 *

*p* < 0.05 *, *p* < 0.01 **, *p* < 0.001 ***. (RBC, Red Blood Cell Count; Hb, Hemoglobin; HCT, Hematocrit; PLT, Platelet; MPV, Mean Platelet Volume).

**Table 3:** Changes in relevant blood biochemical indicators after injection of labeled *Naja atra* venom and labeled *Deinagkistrodon acutus* venom. Marked *Naja atra* venom and *Deinagkistrodon acutus* venom groups showed significantly elevated ALT and AST levels, indicating severe liver damage. After injection with *Deinagkistrodon acutus* venom, fibrin expression could not be detected in the blood, but this was not the case with marked *Naja atra* venom (n = 6).

Project	Control	Cy7-SE + *Naja atra*	Cy7-SE + *Deinagkistrodon acutus*
AST	125.456 ± 8.55	265.104 ± 61.577 **	213.083 ± 46.664 *
ALT	44.937 ± 4.9	78.776 ± 16.826 *	142.898 ± 48.888 *
DBIL	6.436 ± 1.996	4.554 ± 0.736	5.48 ± 1.422
CRE	22.838 ± 4.103	41.543 ± 5.688 **	14.89 ± 1.378 *

*p* < 0.05 *, *p* < 0.01 **. (AST, Aspartate aminotransferase; ALT, Alanine Aminotransferase; DBIL, Direct Bilirubin; CRE, Crea).

**Table 4:** Shotgun identified snake venom in the corresponding organs of labeled *Naja atra/Deinagkistrodon acutus* venom at the timepoint of fluorescence high enrichment in mice.

Protein Name	Target Organ	Species
Venom thrombin-like enzyme (Fragment)	Lung	*Deinagkistrodon acutus*
Venom thrombin-like enzyme	Liver	*Deinagkistrodon acutus*
Snake venom metalloproteinase Ac1	Liver	*Deinagkistrodon acutus*
Snake venom serine protease Dav-X	Liver	*Deinagkistrodon acutus*
Venom thrombin-like enzyme (Fragment)	Brain	*Deinagkistrodon acutus*
Thrombin-like enzyme 2	Diaphragm	*Deinagkistrodon acutus*
Zinc metalloproteinase-disintegrin-like agkihagin	Diaphragm	*Deinagkistrodon acutus*
Zinc metalloproteinase-disintegrin-like acurhagin	Diaphragm	*Deinagkistrodon acutus*
Venom thrombin-like enzyme	Spleen	*Deinagkistrodon acutus*
Snake venom metalloproteinase acutolysin-C	Spleen	*Deinagkistrodon acutus*
Acidic phospholipase A2	Spleen	*Deinagkistrodon acutus*
Acidic phospholipase A2 natratoxin	Spleen	*Naja atra*
Probable weak neurotoxin NNAM2	Kidney	*Naja atra*
Probable weak neurotoxin NNAM2	Liver	*Naja atra*
Probable weak neurotoxin NNAM2	Muscle	*Naja atra*

**Table 5:** Relevant Conditions for MaxQuant Database Retrieval.

Item	Value
Enzyme	Trypsin
Max Missed Cleavages	2
Fixed modifications	Carbamidomethyl (C)
Variable modifications	Oxidation (M), Acetyl(Protein N-term)
Database	uniprot_mouse_76417
Peptide FDR	≤0.01
Protein FDR	≤0.01

**Table 6:** Proteome Discoverer Identification Conditions.

Item	Value
Enzyme	Trypsin
Max Missed Cleavages	2
Fixed modifications	Carbamidomethyl (C)
Variable modifications	Oxidation (M), Acetyl(Protein N-term)
Database	uniprot_mouse_76417
FDR	≤0.01

## Data Availability

The original contributions presented in this study are included in the article material. Further inquiries can be directed to the corresponding author(s).
